# Traditional herbal medicine therapy of gallbladder ascariasis: a case report

**DOI:** 10.1186/s13256-020-02571-x

**Published:** 2021-01-30

**Authors:** Zordun Israyil, Aman Gul, Nassirhadjy Memtily, Aierken Abulizi, Gheni Emet, Batur Niyaz

**Affiliations:** 1 Department of Surgery, Hospital of Xinjiang Traditional Uyghur Medicine, Urumqi, 830049 China; 2 Central Laboratory, Xinjiang Medical University, Urumqi, 830011 China; 3 Department of Integrative Medicine, Huashan Hospital, Fudan University, 12 Middle Urumqi Road, Shanghai, 200040 China; 4 Institute of Theories and Application, Institute of Integrative Medicine, Fudan University, 12 Middle Urumqi Road, Shanghai, 200040 China; 5 Traditional Uyghur Medicine Institute, Xinjiang Medical University, Urumqi, 830011 China; 6 The Imaging Center, The First Affiliated Hospital of Xinjiang Medical University, Urumqi, 830054 China

**Keywords:** Gallbladder ascariasis, Sirkenjibin buzuri, Dinar sherbiti, Kasin jewhiri, Magnesium sulphate

## Abstract

**Background:**

Ascariasis is one of the common intestinal infections in developing countries, including China. Migration of Ascaris lumbricoides into the gallbladder is rare, unlike ascariasis of the bile duct and when it does occur, treatment is generally by endoscopic or surgical extraction.

**Case presentation:**

A 4-year-old Uyghur boy with a history of ascariasis developed intermittent upper abdominal pain for 7 days, was being treated by a local practitioner, and when the pain worsened with yellow sclera for 3 days, he was admitted to our hospital. On physical examination, found out the patient with yellowish skin tone, pale yellow fur on tongue, mild yellow staining of the sclera and tenderness in epigastrium. Laboratory data plus liver function test showed damage of liver function. Abdominal Ultrasonography (USG) and Magnetic resonance cholangiopancreatography (MRCP) showed a long, linear, echogenic structure in the gallbladder neck near to the common bile duct. Once the ascariasis diagnosis was established, he was given conservative treatment of magnesium sulfate with herbal medicine. In 4 days, the patient discharged Ascaris through the stool.

**Conclusions:**

Conservative treatment of magnesium sulfate with Uyghur medicine treatment according to syndrome differentiation is proven to have curative effect.

## Background

Ascaris lumbricoides, Helminths or roundworm, is considered as the most common parasitic infection worldwide. The adult form of Helminths usually resides in the human intestinal lumen (most frequently in the jejunum and middle ileum) and does not cause symptoms [1, 2]. Migration of the worm into the biliary tree is not uncommon and is considered to be a frequent complication of intestinal ascariasis [3, 4]. Gallbladder involvement is very rare, it leads to 2.1% of all biliary tract ascariasis cases [1, 4]. Ultrasonography (USG) is the most commonly used diagnostic modality for this pathology [5, 6]. Computed tomography (CT) magnetic, resonance imaging (MRI) and endoscopic retrograde cholangiopancreatography (ERCP) are also useful [7]. Most of the patients were treated through endoscopy or surgery [8–10]. There are a few reports of conservative medical treatment [11–15]. In this article, we present the radiologic findings, clinical manifestations and successful herbal medicine treatment of a patient with gallbladder ascariasis (GA).

## Case presentation

**Fig. 1:**
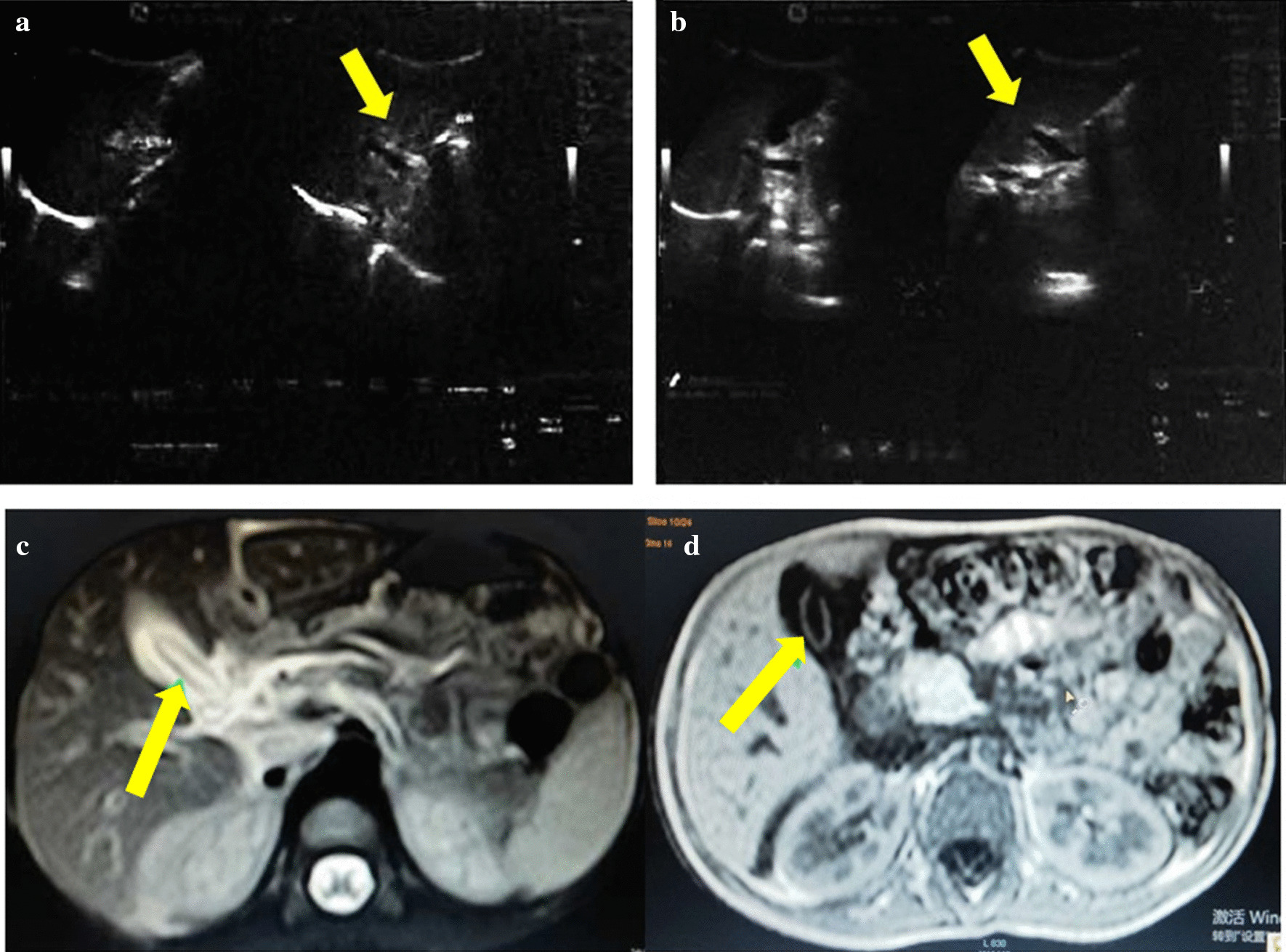
Ultrasonography and Magnetic resonance cholangiopancreatography image of Gallbladder ascariasis. **a** USG image showed structures (arrows) with hypoechogenic central and hyperechogenic edges with no acoustic shadow in the gallbladder suggested ascariasis. **b** There was no abnormal gallbladder size, and no abnormal echo in it, suggested no ascariasis in it. **c**, **d** T2-weighted axial magnetic resonance image showed a linear, hypointense band in the gallbladder (arrows), suggested AG.

A 4-year-old Uyghur boy suffered from intermittent pain in upper abdomen for 7 days, treated by a local practitioner with anthelmintic drugs; however, the pain worsened with yellow sclera for 3 days, then he was admitted to our hospital on October 3, 2016. The boy had a history of roundworm disease nearly a year ago and recovered after a combination treatment with pumpkin seeds and anti-ascaris drugs. On physical examination, we found him moderate nutrition, moderate subcutaneous lipid barrier, medium body size, yellowish skin tone, slightly lower skin temperature, slightly dry skin, red tongue with pale yellow fur, mild yellow staining of the sclera, no yellow staining of the skin and mucous membranes. Furthermore, tenderness was found in epigastrium, the rest of abdomen was soft and non-tender. Bowel sound was normal, no lump palpable. Laboratory data showed (Oct 3, 16.): eosinophils 0.02 × 10^9^; stoll ova and parasite (O&P) test was negative; total bilirubin (TBIL): 128.57 umol/l, direct bilirubin (DBIL): 116.38 umol/l, Alanine aminotransferase (ALT): 2122 u/l, Aspartate aminotrans-ferase (AST): 389 u/l, Gamma glutamyltransferase (GGT): 209 u/l, Serum total bile acid (TBA): 214 u/l, Cholinesterase (CHE): 5057 u/l, Adenosine deaminase (ADA): 26.30 u/l, respectively. Abdominal USG showed a long, linear, moving echogenic structure in the gallbladder, but no abnormal dilation of the bile duct (Fig. 1a). Abdominal magnetic resonance cholangiopancreatography (MRCP) showed liver volume was normal, no obvious abnormal signals was seen in the liver parenchyma, the intrahepatic bile duct and common bile duct were unclear, the gallbladder volume was enlarged, the wall was rough, with curved (linear) long signals more obvious than USG image(Fig. 1c, d). After the diagnosis was established, our patient was given conservative treatment of magnesium sulfate 30 ml/qd with TUM Sirkenjibin buzuri(SB) 10 ml/tid (Sirikanjiben buzure heji, Sirkenjibin buzuri mixture, Xinjiang Drug Approval No. M20041575), Dinar sherbiti(DS) 50ml/tid (Xiaoyan dinaer tangjiang, Dinar syrup, Xinjiang Uygur Pharmaceutical Co., Ltd. Z65020183), Kasin Jewhiri(KJ) 6 g/tid (Qingre kasen keli, Kasen granules, Xinjiang Uyghur Pharmaceutical Co., Ltd. Z65020172). In 4 days, the patient discharged roundworms through the stool. After 5 days, repeated USG showed disappearance of Ascaris from the gall bladder and common bile duct (Fig. 1b).

### Follow-up

The follow up monitoring of the patient was carried out. On 04 October, 2016, test repeated report see Table 1. Other test results were urine specific gravity 1020, pH 6.5, protein nil, blood nil, pus cells nil, cast nil, crystals nil, bacteria nil, patient was evaluated clinically and BP was 90/60 mm Hg, no lumber pain, no edema, urine output was normal all other complains were also normal. Whereas on 7 October and 16 October 2016, 20 April 2017, test repeated report also see Table 1. Other test results were urine specific gravity 1.015, pH 6.0, albumin negative, blood nil, red blood cell (RBC), casts, crystals were nil, bacteria (−) reported. Laboratory test values are summarized in Table 1.

**Table 1:** Results of laboratory test before and after treatment

Test	T0 (Oct 3, 16)	T1 (Oct 4, 16)	T2 (Oct 7, 16)	T3 (Oct 16, 16)	T4 (Apr 20, 17)	Normal range
TBIL	128.57	139.34	73.41	27.31	18.00	2–25 umol/l
DBIL	116.38	124.09	22.24	19.31	6.50	0–8 umol/l
ALT	2122	1417	560	71.0	38.00	5–40 u/l
AST	389	264	155	52	32.00	8–40 u/l
GGT	209	236	300	169	42	0–30 u/l
TBA	214	205	70	4	8.00	0–12 u/l
CHE	5057	5021	5059	8490	5235	5120–11550 u/l
ADA	26.30	43.45	46	14.25	8.00	4–22 u/l
CREA		28	26			40–135 umol/l
Urinary bilirubin		≥ 103 (3+)	(−)	(−)	(−)	(−)

## Discussion

Unlike bile duct ascariasis, Ascaris rarely enters the gallbladder, once ascariasis occurs; it is usually treated by endoscopy or surgical removal. We describe a case of successful conservative treatment of GA in a 4-year-old boy. The treatment method of Uyghur herbal preparations based on syndrome differentiation has practicality and curative effect, which suggests that it may treated GA with liver protection, choleretic, anti-inflammatory and open obstructive effects. Early combination therapy can help optimize management and avoid preventable complications of GA.

Ascaris lumbricoides, an intestinal roundworm, is one of the most common helminthic human infections worldwide; it infects more than 1 billion people worldwide. Infections are mostly asymptomatic, adult Ascaris worms can survive in the human intestines for over 1–2 years [16–18]. The most common presentation is small bowel obstruction due to the mass of worms which obstructs the lumen of the intestine [16–18]. The most common settlement of the worm is in the jejunum middle of small intestine [16]. Due to the narrow and tortuous structure of the biliary tract, it is rare for the Helminth to invade into the gallbladder, constituting 2.1% of hepatobiliary ascariasis [4]. GA may result in severe complications such as ascending cholangitis, acute acalculous cholecystitis, obstructive jaundice, empyema, pericholecystic abscess, pancreatitis, liver abscesses and septicemia [19–21]. The worm can enter the biliary tree from the duodenum and causes variable symptoms such as biliary colic and obstructive jaundice [1, 2].

USG is an important non-invasive diagnostic procedure in the evaluation of the patients who usually present with a clinical picture suggesting gallstone disease [5, 6, 22]. USG appearance of biliary ascariasis is well documented. The worm is usually seen as a long, linear or curved, no shadowing echogenic strip, containing a central, longitudinal anechoic tube, probably representing the digestive tract [5–8]. Movement of worms in the biliary tree is characteristic and is confirmatory evidence in USG diagnosis [5, 6, 22]. Javid *et al*. reported [2] that 47 of 56 patients with GA were diagnosed by USG and 9 were unable to be diagnosed by USG. After surgery, GA was confirmed, all of which were dead roundworms. USG readily show the movement of the worm in the biliary tree and this is an advantage of USG over CT and MRI [5–7], which is supported by Cha *et al.* [11].

**Table 2:** Ingredients, treatment effects, uses, preparations of Sirkenjibin buzuri, Dinar sherbiti and Kasin jewhiri

Herbal formula	Scientific name	Parts used in TUM	Treatment effects and uses	Preparation	References
Sirkenjibin buzuri (SB)	*Cichorium intybus Linn.* 40 g	Seed	Open liver obstruction, decomposing fat in liver, increasing liver function, tonifying liver and stomach, relieving pain, dieresis and cholagogic effect; used for cholecystitis, hepatitis, hyperlipidemia and dyspepsia	All the herbs were chopped into small pieces and ground. The powdered material (240 g) was in distilled water and boiled three times, each time for 2 hours. Combine the decoction, filter, and concentrate the filtrate to the appropriate amount. Add 400 ml of grape vinegar, add water to adjust the total amount to 1000 ml, and mix well	[23–25]
	*Foeniculum vulgare Mill. *40 g	Seed			
	*Apium graveolence Linn.* 40 g	Seed			
	*Nardostachys jatamansi DC.* 120 g	Seed			
	*Grape vinegar* 400 ml	Vinegar			
Dinar sherbiti (DS)	*Cichorium intybus Linn.* 30 g	Seed	Diuresis, detumescence, antiphlogistic, antipyretic and analgesic effects; used for hepatitis, cholecystitis, urinary tract infection and hyperlipidemia	All the herbs were chopped into small pieces and ground. The powdered material (210 g) was in distilled water and boiled three times, each time for 2 hours. Combine the decoction, filter, and concentrate the filtrate to the appropriate amount. Add 600 g of sucrose, boil to dissolve, filter, add water to adjust the total amount to 1000 ml, and mix well	[26, 27]
	*Cichorium intybus roots* 30 g	Root			
	*Rosepetals* 30 g	Dried petals			
	*Rheum palmatum Linn. *30 g	Roots, rhizomes			
	*Nymphaea candida Presl.* 30 g	Dried buds			
	*Anchusa Italica Retz.* 30 g	aerial part			
	*Cuscuta chinensis Lam. *30 g	Seed			
Kasini jewhiri (KJ)	*Cichorirum intybus *1000 g	Aerial part	Clearing liver toxic materials, promoting digestion, cholagogic, diuresis and detumescence effects; used for hepatitis, nephritis, enteritis, tracheitis	The powdered material (1000 g) add water and boiled for three times, 1.5 hours each time, combined the decoction, filtered, the filtrate was concentrated to a thick paste with a relative density of 1.35 to 1.38 (50 to 60 °C), add an appropriate amount of sucrose and β-cyclodextrin, and mixed well. It was made into granules, dried and made into 1000 g	[28, 29]

Therefore, patients are usually treated by endoscopy or surgery. In this patient, epigastric and right upper quadrant pain with yellow sclera was the main symptom, which is considered to be due to the acute cholecystitis. Contrary to our expectations, there was moderate biliary tree dilatation in this case. The patient had a history of Ascaris in the past year, and his parents reported that he had been cured by pumpkin seeds combined with anti-Ascaris drugs. This time, there were intermittent right upper abdominal pain, jaundice, liver function decline and other clinical manifestations. USG indicates that there was linear substance without curling movement in gallbladder. MRCP reported that there was linear substance in the gallbladder. Combined with the history, it was diagnosed as GA.

Eosinophils slightly increased, O&P experiment was negative, and Ascaris had no movement during the USG examination. GA responds poorly to medical treatment because less than 1% of the antihelminthic drugs are excreted in bile. After the worms migrate out of the biliary tree into the duodenum, the antihelminthic drugs can act upon them [2, 4]. Considering the dead Ascaris, we chose conservative treatment. In this case, we administered combined therapy magnesium sulfate with SB, DS, and KJ [23–29]. Ingredients, treatment effects, uses, preparations of these herbals are summarized in Table 2. The purpose of the combined treatment is to protect the liver, promote the contraction of the gallbladder, change the suitable environment for Ascaris survival, and eliminate it [23–26]. Inoue *et al.*, found that after oral administration, magnesium sulfate can reflexively cause the relaxation of the sphincter muscles of the common bile duct and the contraction of the gallbladder, promote the emptying of the gallbladder and produce a cholagogic effect, which is conducive to the expulsion of Ascaris from the bile duct [30]. After 4 days combined treatment, and we found dead Ascaris bodies excreted from stool. We reviewed by USG the roundworm was found to disappear from the gallbladder (Fig. 1b).

In this case, we continued combined therapy for 10 days. Magnesium sulfate didn't cause diarrhea, dehydration and other adverse reactions. No adverse reactions were found with treatment of SB, DS and KJ. It leads us thought that magnesium sulfate combined with SB, DS and KJ may eliminate Ascaris through contraction of gallbladder, cholagogue blockage [23–30]. Also recover the liver function by anti-inflammation, cholagogue, diuresis and relieving pain effects [23–29].

## Conclusions

Magnesium sulfate combined Uyghur herbal preparations have resolved GA within 4 days, as analyzed by USG, serum and urine tests. Further follow up for another 10 days showed sustained improvement. The early combined treatment can help to optimize management and avoid the preventable complications of GA. Based on syndrome differentiation, the herbal medicine treatment has applicability and curative effect; which indicate liver-protecting, choleretic, anti-inflammatory and open obstruction effect.

In sum up, the results of the study show that there may an effect of above medicine in the treatment of GA. Magnesium sulfate combined Uyghur herbal preparations can improve clinical curative effect of GA, which provides a new way of thinking to clarify herbal preparation’s molecular mechanism and potential curative target not only for the treatment of GA, but also for biliary ascariasis.

## Data Availability

All data used during the present study are available from the corresponding author on reasonable request.

## Abbreviations

TUM: Traditional Uyghur medicine
GA: Gallbladder ascariasis
SB: Sirkenjibin buzuri
DS: Dinar sherbiti
KJ: Kasin jewhiri
O&P test: Stool ova and parasites test
USG: Ultrasonography
CT: Computed tomography
MRI: Magnetic resonance imaging
MRCP: Magnetic resonance cholangiopancreatography
ERCP: Endoscopic retrograde cholangiopancreatography
TBIL: Total bilirubin
DBIL: Direct bilirubin
ALT: Alanine aminotransferase
AST: Aspartate aminotransferase
GGT: Gamma-glutamyltransferase
TBA: Serum total bile acid
CHE: Cholinesterase
ADA: Adenosine deaminase
CREA: Serum creatinine

## References

[1] Taydaş O, Özdemir M, Akyüz B, *et al*. Gallbladder ascariasis: a case report and review of the literature. J Sakarya Med. 2019;9(1):175–9.

[2] Javid G, Wani N, Gulzar GM, *et al*. Gallbladder ascariasis: presentation and management. Br J Surg. 1999;86:1526–7. 10.1046/j.1365-2168.1999.01289.x 10594499

[3] Khuroo MS, Rather AA, Khuroo NS, *et al*. Hepatobiliary and pancreatic ascariasis. World J Gastroenterol. 2016;22:7507–17. 10.3748/wjg.v22.i33.7507 PMC5011666 27672273

[4] Hefny AF, Saadeldin YA, Abu-Zidan FM. Management algorithm for intestinal obstruction due to Ascariasis: a case report and review of literature. TJTES. 2009;15(3):301–5. 19562557

[5] Maierhaba T, Zhang S. Analysis of doppler ultrasound for diagnosis of infantile biliary tract roundworm disease. J China Modern Doctor. 2012;50(31):89–90.

[6] Mahmood T, Mansoor N, Quraishy S, *et al*. Ultrasonographic appearance of Ascaris lumbricoides in the small bowel. J Ultrasound Med. 2001;20:269–74. 10.7863/jum.2001.20.3.269 11270532

[7] Arya PK, Kukreti R, Arya M, *et al*. Magnetic resonance appearance of gall bladder ascariasis. Indian J Med Sc. 2005;59:208–10. 15985729

[8] Goyal A, Gamanagatti S, Sriram J. Tube with in tube: ascaris in bowl and biliary tract. Am J Trop Med Hyg. 2010;83(5):962. 10.4269/ajtmh.2010.10-0358 PMC2963952 21036820

[9] Shah OJ, Zargar SA, Robbani I. Biliary ascariasis: a review. World J Surg. 2006;30:1500–6. 10.1007/s00268-005-0309-1 16874446

[10] Keating A, Quigley JA, Genterola AF. Obstructive jaundice induced by biliary ascariasis. BMJ Case Rep. 2012; 007250. 10.1136/bcr-2012-007250 PMC4544173 23239771

[11] Cha DY, Song IK, Choi HW, *et al*. Successful elimination of Ascaris lumbricoides from the gallbladder by conservative medical therapy. J Gastroenterol. 2002;37:758–60. 10.1007/s005350200125 12375153

[12] Ismaili-Jaha V, Toro H, Spahiu L, *et al*. Gallbladder ascariasis in Kosovo-focus on ultrasound and conservative therapy: a case series. J Med Case Rep. 2018;12:8. 10.1186/s13256-017-1536-4 PMC5767008 29329599

[13] Osman S. Clinical observation on 28 cases of biliary ascariasis treated by the combination of traditional Uyghur and modern medicine. Chin J Ethnomed Ethnopharm. 2012;21(20):99–99.

[14] Alhamid A, Aljarad Z, Ghazal A, *et al*. Case report successful elimination of gallbladder ascariasis by conservative therapy, followed by cholecystectomy due to developing cholecystitis. Case Rep Gastrointest Med. 2018. 10.1155/2018/5831257. PMC6311234 30643654

[15] Misra MK, Singh S, Bhagat TS. Successful elimination of ascaris lumbricoides from the gallbladder by conservative medical therapy. Indian J Surg. 2013;75(S1):379–81. 10.1007/s12262-012-0527-3. PMC3693243 24426622

[16] Centers for Disease Control and Prevention. Parasites-ascariasis. 2020. https://www.cdc.gov/parasites/ascariasis/.

[17] Uysal E, Dokur M. The helminths causing surgical or endoscopic abdominal intervention: a review article. Iran J Parasitol. 2017;12(2):156–68. PMC5527025 28761475

[18] Keshvala C, Naidu L. Not everything in the gallbladder is gallstones: an unusual case of biliary ascariasis. BJR Case Rep. 2019;5(4):20180123. 10.1259/bjrcr.20180123 PMC6945261 31938556

[19] Wang J, Pan YL, Xie Y, *et al*. Biliary ascariasis in a bile duct stones removed female patient. World J Gastroenterol. 2013;19:6122–4. 10.3748/wjg.v19.i36.6122 PMC3785638 24106417

[20] Ibrarullah M, Mishra T, Dash AP, *et al*. Biliary ascariasis–role of endoscopic intervention. Trop Gastroenterol. 2011;32:210–3. 22332337

[21] Lamberton PHL, Jourdan PM. Human ascariasis: diagnostics update. Curr Trop Med Rep. 2015;2:189–200. 10.1007/s40475-015-0064-9 PMC4630244 26550552

[22] Alam S, Mustafa G, Rahman S, *et al*. Comparative study on presentation of biliary ascariasis with dead and living worms. Saudi J Gastroenterol. 2010;16(3):203–6. 10.4103/1319-3767.65200. PMC3003205 20616417

[23] Hemdul A, Osman E. Clinical research report of Sirkenjibin Buzuri. Women’s Health Res. 2015;13:235.

[24] Zhang C, Zhang K, Tian X, *et al*. Hepatoprotective effect of Hugan buzure keli on carbon tetrachloride induced-hepatic fibrosis in mice. J Shihezi Univ (Natl Sci). 2016;34(3):355–9.

[25] Kurban A. Treatment of 20 cases of fatty liver with TUM Sirkenjibin buzuri. J Med Pharm Chin Minor. 2011;4:9–10. 10.16041/j.cnki.cn15-1175.2011.04.009.

[26] Nur H, Semet R, Eemet M, Gheni S. Clinical research report of TUM Dinar sherbiti. J Med Pharm Chin Minor. 2011;6:7–8. 10.16041/j.cnki.cn15-1175.2011.06.009.

[27] Shakya AK, Shukla S. Protective effect of Sharbat-e-Deenar against acetaminophen-induced hepatotoxicity in experimental animal. J Tradit Chin Med. 2017;37(3):387–92. 31682382

[28] Neha M, Deepshikha KP, Vidhu A. *Cichorium intybus* Linn: its role in hepatoprotection. Int J Pharmacogn Phytochem Res. 2014;6(3):499–505.

[29] Memtimin G, Abdurahman G. Observation on 61 cases of fatty liver treated with TUM Kasin Jewhiri. J Med Pharm Chin Minor. 2014;1:12–3. 10.16041/j.cnki.cn15-1175.2014.01.011.

[30] Inoue K, Wiener I, Fagan CJ, *et al*. Correlation between gallbladder size and release of cholecystokinin after oral magnesium sulfate in man. Ann Surg. 1983;197(4):412–5. 10.1097/00000658-198304000-00006 PMC1352753 6830347

